# The effect of social support around pregnancy on postpartum depression among Canadian teen mothers and adult mothers in the maternity experiences survey

**DOI:** 10.1186/1471-2393-14-162

**Published:** 2014-05-07

**Authors:** Theresa HM Kim, Jennifer A Connolly, Hala Tamim

**Affiliations:** 1 School of Kinesiology and Health Science, York University, 4700 Keele Street, Toronto, Ontario M3J 1P3, Canada; 2 Department of Psychology, York University, 4700 Keele Street, Toronto, Ontario M3J 1P3, Canada

**Keywords:** Social support, Postpartum depression, Teenage pregnancy

## Abstract

**Background:**

Postpartum depression (PPD) is a mood disorder that affects 10–20 percent of women, and can begin any time during first year after delivery lasting for months. Social support may decrease risk of depression during pregnancy for women. However, literature shows that the amount of social support received during and after pregnancy is different for teen mothers and adult mothers. This study examined the effects of social support received during and after pregnancy on PPD among Canadian women and identified if the relationship was different for teen mothers compared to adult mothers.

**Methods:**

The study was based on secondary analysis of the Maternity Experiences Survey. A total of 6,421 women with singleton live births, aged 15 years and older were analyzed. Teen mothers were identified as 15–19 years old and adult mothers were identified as 20 years and older. The main outcome of the study was PPD, which was evaluated using the Edinburg Postnatal Depression Scale. The main independent variable was social support received during pregnancy and after birth. Logistic regression was computed to assess the relationship between social support and PPD after adjusting for confounding variables and age as an interaction term. Adjusted Odds Ratios and 95% Confidence Intervals were reported.

**Results:**

PPD was experienced by 14.0% among teen mothers and 7.2% among adult mothers (p < .001). Overall, teen mothers reported receiving more support during pregnancy and after birth than adult mothers (p < .010). The relationship between social support and PPD did not significantly differ for teen compared to adult mothers. Both teen and adult mothers were approximately five times more likely to experience PPD if they received no support or minimal support after the birth of the baby (95% CI, 3.51-7.36).

**Conclusion:**

Receiving social support especially after birth is important for mothers of all ages to reduce the risk of PPD.

## Background

Social support is a multi-faceted complex concept that has been extensively studied to define and measure. Broadly defined, social support is a voluntary act from one individual (the donor) that is given to another individual (the recipient), which elicits an immediate or delayed positive response in the recipient [1]. The voluntary act can be given by a family member, friend, husband/partner, and/or others, and it may be given in different forms: informational, physical, emotional (e.g. empathy, caring, love), instrumental (e.g. financial), and appraisal (e.g. information promoting self-evaluation) [2]. Groundbreaking research from the 1970s has shown that the social environment has a direct benefit to health outcomes, such that social support was shown to prevent disease [3]. The consensus of research in the past thirty years have shown that individuals who have greater social support are at odds of greater longevity than those with fewer social ties [4]. Research regarding the association between social support and health moved from mortality risk to morbidity risk, affecting pregnancy outcomes such as fetal (e.g. preterm birth), infant (e.g. low birth weight), and maternal health (e.g. postpartum depression).

Postpartum depression (PPD) is a mood disorder that affects 10–20 per cent of women, and can begin any time during first year after delivery lasting for months [5,6]. Its symptoms include sadness, fatigue, changes in sleeping and eating patterns, reduced libido, crying episodes, anxiety, irritability, feelings of loneliness, emotional lability, and even thoughts of harming oneself and/or the child [6]. Some significant risk factors for PPD include smoking [7,8], low levels of social support [9], low socioeconomic status, obstetric complications, and stressful life events during pregnancy [10]. Postpartum depression is associated with self-inflicted injury and morbidity for the mother [10] and poorer responsiveness to infant cues and disengaged parenting behaviours that ultimately lead to harmful cognitive and emotional development in infants [11].

Evidence has shown that social support may decrease risk of depression during pregnancy, which leads to positive health and pregnancy outcomes [4,12-15]. The level of social support received around pregnancy is different for teen mothers (15–19 years old) and adult mothers (20+ years old) [12,13]. Teen mothers reported receiving significantly less social support than adult mothers as they had poorer ability to make and maintain relationships with others [12,13]. It was observed that teen mothers were more deprived in terms of lower education, lower social class (higher unemployment rate), and were more likely to be single compared to adult mothers [12,13]. Studies have indicated that mothers who received social support during pregnancy and/or after childbirth were significantly less likely to experience PPD [16-18]. However, theses studies were either limited in generalizability, observed only teen mothers, did not include age of mother, or observed only social support received during pregnancy [16-18].

Other studies using the Maternity Experiences Survey have examined PPD as a function of outcome among mothers. Recent study by Kingston, Heaman, Fell, and Chalmers examined the maternity experiences and behaviours among three groups of mothers – adolescent mothers, young adult mothers, and adult mothers, and found that adolescent and young adult mothers were more likely to experience PPD [19]. Vigod and colleagues examined area of residence and risk of PPD [20], and found that prevalence of PPD was greater among mothers living in urban areas compared to rural areas. An epidemiology study by Dennis and colleagues determined that low postpartum support was a significant indicator of PPD among Canadian mothers [21]. However, in all these studies, the relationship between social support and PPD in the context of observing the differences in teen mothers and adult mothers has not been examined.

The purpose of this study was to examine the association of social support received during pregnancy and after childbirth on PPD in a nationally representative sample of Canadian women and identify if the relationship is different for teen mothers (15–19 years old) compared to adult mothers (20+ years old).

## Methods

### Study design

The current study was a cross-sectional study as the analysis was based on secondary data analysis. The database used was the Maternity Experiences Survey.

### Database

The database used in the analysis to address the research objectives was the Maternity Experiences Survey (MES), a project of the Public Health Agency of Canada in collaboration with Statistics Canada. The MES is a Canadian nationwide cross-sectional survey that collected information on women’s perceptions, knowledge, and practices 12 months prior to pregnancy, during pregnancy, and after childbirth, focusing on the determinants of maternal, fetal, and infant health. The MES identified a stratified random sample of 8,244 women using the 2006 Canadian Census, of which 6,421 (75.2%) women completed the interview. Eligibility criteria included women who gave singleton live births between the period of November 2005 and May 2006, aged 15 years and older, and who lived with their infant at the time of the interview. The interviews were conducted via computer-assisted telephone or face-to-face at five to 14 months after the birth of their baby. The majority (96.9%) of the interviews were conducted between 5^th^ and 9^th^ month after delivery, and they were approximately forty-five minutes long. The MES database was presented to Health Canada’s Science Advisory Board, Health Canada’s Research Ethics Board and the Federal Privacy Commissioner and was approved by Statistics Canada’s Policy Committee. The data was accessed through an application process to the Social Sciences and Humanities Council of Canada. Permission was granted to access the database through the Research Data Centre in Toronto. The MES has been previously described elsewhere [22].

### Measures

#### Outcome variable

The main outcome variable of the study was postpartum depression, which was evaluated using the Edinburgh Postnatal Depression Scale (EPDS) [23]. The EPDS is a validated 10-item screening tool [23], and is the most widely used screening questionnaire for PPD. A total score can range from 0 (indicating no risk for postpartum depression) to 30 (indicating postpartum depression) [24]. This screening tool was administered to all survey participants. Dichotomous scoring was created to determine postpartum depression. A score ranging from 0–13 were recoded as “no postpartum depression”, and a score ranging from 14–30 were recoded as “yes postpartum depression”.

#### Independent variable

Social support received during pregnancy and social support received after childbirth were the main independent variables in this study. In the MES database, social support was assessed by the question “how often was support available to you when you needed it?” Responses to the question were: none of the time, little of the time, some of the time, most of the time, all of the time. The response categories were combined to three categories; minimal support (combining support received none, little, or some of the time); most of the time; and all of the time.

#### Covariates

Covariate variables that were considered in the analyses included: *socio-demographic factors* (age, place of residence, immigration, education, partner status, previous depression diagnosis), *pregnancy-related characteristics* (wanting the pregnancy, health problems during pregnancy, working during pregnancy, smoking during third trimester of pregnancy, alcohol use during third trimester of pregnancy, drug use during third trimester of pregnancy), *delivery characteristics* (type of delivery, birth setting, general experience in labour and birth), and *postpartum characteristics* (mother’s hospitalization after birth, working after pregnancy, receiving enough information on PPD). All of these variables were directly self-reported by the mother.

### Statistical analysis

Chi square-test was performed to assess significant differences between social support, PPD, socio-demographic, pregnancy-related, delivery, and postpartum characteristics comparing teen mothers aged 15–19 years to adult mothers aged 20 years and older. For the bivariate analysis, the relationship between the independent variables and PPD were assessed using chi-square tests, reporting Odds Ratios (OR) and 95% confidence intervals (95% CI). Logistic regression model was performed to assess the independent relationship between social support and PPD adjusting for age of the mother (teen mothers aged 15–19 years and adult mothers aged 20 years and older) and all other variables. Two interaction terms; one for age of the mother and social support during pregnancy and one for age of the mother and social support after birth were included in the model to assess the significance of the interaction of age with the relationship of social support and PPD. Adjusted ORs and 95% CI were reported. To account for the complex sampling design, bootstrapping was performed to calculate all the 95% CI estimates [25]. Population weights, normalized weights and bootstrap weights were all created by Statistics Canada and provided with the MES data set. All the analyses, except for bootstrapping, were computed with the Statistical Package for Social Sciences (SPSS, version 17.0). Bootstrapping was performed using Stata Data Analysis and Statistical Software (version 10.0). All analyses was set at alpha <0.05 for two tailed test for statistical significance.

## Results

The sample size of 6,421 was analyzed in this study, which was weighted to represent 76,508 Canadian women. Out of 6,421 Canadian women, 6,304 had complete information on PPD and thus were included in the analysis. A total of 288 were teen mothers aged 15–19 years.

Overall, 14.0% of teen mothers and 7.2% of adult mothers experienced PPD (p < 0.01). Teen mothers received significantly more support during pregnancy than adult mothers (65.0% vs. 57.4% respectively, p = 0.035) as well as after birth (60.4% vs. 51.0% respectively, p = 0.003). During pregnancy, 65.0% of teen mothers and 57.4% of adult mothers reported receiving support all of the time. Similarly, after birth, 60.4% of teen mothers and 51.0% of adult mothers reported receiving support all of the time. Majority of adult mothers reported having a partner throughout their pregnancy (92.9%) compared to only 51.8% of teen mothers (p < 0.001). Majority of mothers reported receiving enough information about PPD (89.5% among teen mothers and 92.1% among adult mothers, p = 0.174). The characteristics for teen mothers and adult mothers are shown in Table 1.

**Table 1:** Overall characteristics of Canadian teen mothers and adult mothers in 2006–2007 maternity experiences survey (N = 6,304)

**Characteristics**	**Teen mothers**	**Adult mothers**	**All mothers**	**P-value‡**
	**N (%)**	**N (%)**	**N (%)**	
***Outcome variable***				
**Postpartum depression**				<0.001
No	160 (86.0)	5,676 (92.8)	5836 (92.6)	
Yes	26 (14.0)	442 (7.2)	468 (7.4)	
***Social support around pregnancy***				
**Overall support during pregnancy**				0.035
All of the time	187 (65.0)	3,443 (57.4)	3630 (57.7)	
Most of the time	67 (23.1)	1,791 (29.8)	1858 (29.5)	
None/Little/Some of the time	34 (11.9)	768 (12.8)	802 (12.8)	
**Overall support after birth**				0.003
All of the time	173 (60.4)	3,067 (51.0)	3240 (51.5)	
Most of the time	68 (23.7)	1,997 (33.3)	2065 (32.8)	
None/Little/Some of the time	46 (15.9)	943 (15.7)	989 (15.7)	
***Socio-demographic characteristics***				
**Age**				
Teen mother (15–19 years)	N/A	N/A	186 (3.0)	
Adult mother (20+ years)	N/A	N/A	6118 (97.0)	
**Urban–rural residence**				<0.001
Rural area	51 (18.8)	1,042 (17.9)	1093 (17.9)	
Urban, population ≤499,999	136 (50.2)	2,145 (36.8)	2281 (37.4)	
Urban, population ≥500,000	84 (31.0)	2,644 (45.3)	2728 (44.7)	
**Immigration to Canada**				
No	N/A	N/A	4953 (78.4)	
Yes	N/A	N/A	1362 (21.6)	
**Level of education**				
High school or less	N/A	N/A	1307 (20.8)	
Some postsecondary education	N/A	N/A	2743 (43.6)	
Undergraduate education	N/A	N/A	1617 (25.7)	
Graduate education	N/A	N/A	619 (9.9)	
**Partner/Significant other**				<0.001
No	137 (48.2)	427 (7.1)	564 (9.0)	
Yes	148 (51.8)	5,576 (92.9)	5724 (91.0)	
**Previous depression diagnosis**				
No	N/A	N/A	5350 (84.6)	
Yes	N/A	N/A	977 (15.4)	
***Pregnancy-related characteristics***				
**Wanted the pregnancy**				<0.001
Then or Sooner	77 (27.1)	4,443 (74.3)	4520 (72.2)	
Later or Not at all	207 (72.9)	1,534 (25.7)	1741 (27.8)	
**Health problems during pregnancy**				0.822
No	216 (74.9)	4,536 (75.5)	4752 (75.5)	
Yes	72 (25.1)	1,473 (24.5)	1545 (24.5)	
**Work during pregnancy**				<0.001
No	226 (79.4)	5,450 (90.8)	5676 (90.2)	
Yes	59 (20.6)	555 (9.2)	614 (9.8)	
**Cigarette smoking during pregnancy**				<0.001
No	205 (71.1)	5,411 (90.0)	5616 (89.1)	
Yes	83 (28.9)	601 (10.0)	684 (10.9)	
**Alcohol use during pregnancy**				
No	N/A	N/A	5638 (89.4)	
Yes	N/A	N/A	668 (10.6)	
**Drug use during pregnancy**				
No	N/A	N/A	6263 (99.0)	
Yes	N/A	N/A	63 (1.0)	
***Delivery characteristics***				
**Type of delivery**				0.025
Vaginal	230 (79.8)	4,424 (73.6)	4654 (73.9)	
Caesarean	58 (20.2)	1,586 (26.4)	1644 (26.1)	
**Birth setting**				
Hospital/clinic	N/A	N/A	6207 (98.0)	
Birthing centre/private/other	N/A	N/A	129 (2.0)	
**General experience in labour & birth**				
Negative	N/A	N/A	578 (9.2)	
Positive	N/A	N/A	5058 (80.2)	
Neither	N/A	N/A	671 (10.6)	
***Postpartum characteristics***				
**Mother’s hospitalization after birth**				<0.001
No	265 (92.0)	5,828 (96.9)	6093 (96.7)	
Yes	23 (8.0)	188 (3.1)	211 (3.3)	
**Work after pregnancy**				
No	N/A	N/A	5461 (86.4)	
Yes	N/A	N/A	861 (13.6)	
**Enough information on PPD**				0.174
No	30 (10.5)	478 (7.9)	508 (8.1)	
Yes	257 (89.5)	5,535 (92.1)	5792 (91.9)	

‡p-value calculated using bootstrapping technique.

N/A – Not Available - Data not allowed for release due to cell count less than 5.

Table 2 shows the unadjusted and adjusted associations between social support and PPD among mothers. Results of the multivariate analysis showed no statistical significance for either interaction terms included in the model suggesting that the relationship between social support and PPD is not significantly different for teen mothers in comparison to adult mothers. Hence the data for teen mothers and adult mothers was combined for analysis. No significant association was found between support received during pregnancy and PPD (adjusted OR, 1.31, 95% CI, 0.91-1.91) however; mothers were approximately five times at increased risk of experiencing PPD if they received minimal support after the birth of the baby (adjusted OR, 5.10, 95% CI, 3.51-7.36). Age of the mother was found to be a significant predictor of PPD. Adult mothers aged 20 years and older were 44% less likely to experience PPD (adjusted OR, 0.56, 95% CI, 0.36-0.89).

**Table 2:** Associations between social support and postpartum depression among Canadian mothers in 2006–2007 maternity experiences survey (N = 6,304)

**Independent variables**	**Unadjusted odds ratio**	**Adjusted odds ratio**
	**OR (95% CI)‡**	**OR (95% CI)‡**
***Social support around pregnancy***		
**Overall support during pregnancy**		
All of the time	1.00	1.00
Most of the time	2.25 (1.79-2.82)	1.27 (0.95-1.69)
None/Little/Some of the time	4.39 (3.39-5.68)	1.31 (0.91-1.91)
**Overall support after birth**		
All of the time	1.00	1.00
Most of the time	2.49 (1.90-3.26)	2.11 (1.51-2.95)
None/Little/Some of the time	8.02 (6.17-10.44)	5.10 (3.51-7.36)
***Socio-demographic characteristics***		
**Age**		
Teen mother (15–19 years)	1.00	1.00
Adult mother (20+ years)	0.47 (0.33-0.68)	0.56 (0.36-0.89)
**Urban–rural residence**		
Rural area	1.00	1.00
Urban, population ≤499,999	1.00 (0.73-1.36)	0.98 (0.69-1.37)
Urban, population ≥500,000	1.56 (1.17-2.07)	1.36 (0.99-1.88)
**Immigration to Canada**		
No	1.00	1.00
Yes	2.19 (1.75-2.74)	1.92 (1.44-2.57)
**Level of education**		
High school or less	1.00	1.00
Some postsecondary education	0.60 (0.47-0.75)	0.75 (0.56-1.01)
Undergraduate education	0.54 (0.40-0.72)	0.72 (0.50-1.02)
Graduate education	0.49 (0.32-0.76)	0.54 (0.33-0.88)
**Partner/Significant other**		
No	1.00	1.00
Yes	0.54 (0.41-0.72)	0.77 (0.55-1.08)
**Previous depression diagnosis**		
No	1.00	1.00
Yes	2.46 (1.96-3.08)	2.44 (1.88-3.17)
***Pregnancy-related characteristics***		
**Wanted the pregnancy**		
Then or Sooner	1.00	1.00
Later or Not at all	1.78 (1.45-2.19)	1.23 (0.96-1.58)
**Health problems during pregnancy**		
No	1.00	1.00
Yes	1.56 (1.26-1.94)	1.45 (1.13-1.87)
**Work during pregnancy**		
No	1.00	1.00
Yes	1.43 (1.05-1.93)	1.47 (1.01-2.12)
**Cigarette smoking during pregnancy**		
No	1.00	1.00
Yes	1.61 (1.23-2.11)	1.22 (0.87-1.71)
**Alcohol use during pregnancy**		
No	1.00	1.00
Yes	1.15 (0.83-1.59)	1.10 (0.76-1.59)
**Drug use during pregnancy**		
No	1.00	1.00
Yes	4.89 (2.66-8.98)	2.39 (1.08-5.26)
***Delivery characteristics***		
**Type of delivery**		
Vaginal	1.00	1.00
Caesarean	0.92 (0.74-1.16)	0.90 (0.69-1.16)
**Birth setting**		
Hospital/clinic	1.00	1.00
Birthing centre/private/home/other	1.16 (0.51-2.66)	1.24 (0.50-3.12)
**General experience in labour and birth**		
Negative	1.00	1.00
Positive	0.63 (0.46-0.85)	0.77 (0.55-1.08)
Neither	0.62 (0.40-0.95)	0.73 (0.45-1.21)
***Postpartum characteristics***		
**Mother’s hospitalization after birth**		
No	1.00	1.00
Yes	1.84 (1.17-2.88)	1.66 (0.98-2.82)
**Work after pregnancy**		
No	1.00	1.00
Yes	0.72 (0.52-1.00)	0.81 (0.56-1.16)
**Enough information on PPD**		
No	1.00	1.00
Yes	0.26 (0.20-0.33)	0.38 (0.28-0.51)

‡95% CIs calculated using bootstrapping technique.

OR = odds ratio; 95% CI = 95% confidence interval.

Socio-demographic characteristics such as immigration status, level of education, and previous depression diagnosis was a significant predictor of PPD. Mothers who immigrated to Canada were almost twice as likely to experience PPD (95% CI, 1.44-2.57) and those who had a previous depression diagnosis were more than twice as likely to experience PPD (adjusted OR, 2.44, 95% CI, 1.88-3.17). However, holding a graduate education degree reduced the likelihood of experiencing PPD (adjusted OR, 0.54, 95% CI, 0.33-0.88).

Pregnancy-related characteristics such as health problems during pregnancy, work during pregnancy, and drug use during pregnancy were significant predictors of PPD. Mothers who received enough information about PPD after birth were 62% less likely to experience PPD (adjusted OR, 0.38, 95% CI, 0.28-0.51).

## Discussion

The purpose of this study was to examine the effect of social support received during pregnancy and after childbirth on PPD in a nationally representative sample of Canadian women and to determine if the relationship differed for teen mothers and adult mothers. Results showed that the relationship between social support and PPD is not different for teen mothers and adult mothers after adjusting for confounding variables.

Literature shows that teen mothers are at greater risk for PPD compared to adult mothers [26-28]. Studies have shown that PPD affects as much as 26% among teen mothers in comparison to 13% among the general population [26,28,29], a ratio that is consistent with the present study. The proportional differences in vulnerability to PPD between teen mothers and adult mothers may be partially explained by lifestyle factors and emotional immaturity [27] as teen mothers may lack preparedness for transition into motherhood.

Motherhood requires lots of support in various forms (e.g., financial, appraisal, physical, emotional), and from sources - family, partners, and peers. Generally, adult mothers are known to receive more support regarding their pregnancy compared to teen mothers [12,13]. Contrary to our study results, literature has shown that teen mothers are reported to have poorer ability to build and maintain interpersonal relationships, indicating the absence of any adequate support compared to adult mothers [13]. One can speculate that this may be due to the fact that teen mothers are not able to readily connect with and relate to majority of their peers who are not going through pregnancy and motherhood. Opportunities to build connections and network with peers and colleagues are not as readily available to teen mothers. Teen mothers usually are unable to finish school and hold lower education and lower social class status than adult mothers [12,13,30]. This leads them to be faced with economic burdens as young motherhood may truncate education, which in turn limits opportunities for better employment, and to build and maintain interpersonal relationships [30]. Social support was not defined using a standardized tool in the MES, and is a multi-faceted concept that may be influenced by time and cultural environment. Other factors that were not available in the MES database such as bullying and peer pressure from others may explain and predict risk of PPD even when mothers reported that social support was abundantly received. Needless to say, there is a strong need to further research the cultural-specific definition of social support received among Canadian mothers especially one that draws the distinction between teen mothers and adult mothers.

Results of the present study showed that the relationship between social support and PPD was not significantly different for teen mothers as compared to adult mothers. It suggests that mothers despite their age were at equal risk for PPD when they did not receive any or minimal support after childbirth. Despite the variability in definition of social support, studies have shown that higher levels of support were associated with lower prevalence of PPD among mothers [16-18]. In a study inclusive of women of all ages, Liabsuetrakul et al. [18] found that one of the important and significant post partum predictors of PPD was social support, however, social support during pregnancy was not a significant predictor. In a study by Barnet, et al. [16], the most common support providers for pregnant teens were their mothers and their partners. Teen mothers with high stress and low social support during their pregnancy were at significant risk for depressive symptoms [16]. The absence or lack of support from their partners was eminent among teen mothers as they were more likely to be single (unmarried and/or not living with their partners) [12,13,30]. Similarly, in a study by Turner et al. [17], poor family support and poor friend support were predictors of depression among adolescent mothers. Studies have also found that marital status (i.e. single or married) was not significantly associated with the risk of PPD [17,31], which, the current study also reports. This allows one to speculate that having a partner while pregnant may not be as important, and that specifically looking into partner support will be more crucial for predicting the risk of PPD.

Contrary to literature [7,8,32], lifestyle factors such as smoking during pregnancy and alcohol use during pregnancy did not significantly increase the likelihood of experiencing PPD. However, drug use during pregnancy significantly increased the likelihood of experiencing PPD by twofold. Approximately 10% of women in this population smoked cigarettes and drank alcohol during the pregnancy and about 1% used drugs. Although such a minuscule number of Canadian women reported using drugs during pregnancy, it had a profound effect on the risk of PPD among mothers.

Evidence of this present study suggested that education programs on PPD may prove to be an exceptionally important benefit as it acts as a protective factor for PPD. Recent studies have shown that providing education about PPD after childbirth has shown to be effective in decreasing the risk of depression in mothers [33,34]. In addition, it is more beneficial to give information about PPD to mothers during hospitalization immediately after delivery, as they are more responsive to information about PPD given at that time than in the prenatal stages, as it did not reduce PPD [35-38].

The results found in this study should be cautiously interpreted by taking certain limitations into consideration. Due to the limitation of not being able to release results with cell count ≤5, the following variables were removed from bivariate analysis: immigrant status, level of education, alcohol use, drug use, work status after birth, birth setting, and general experience of labour and birth. Confounding variables that were related to women’s history of depression such as the duration or timing of occurrence, or nature of the treatment received for depression were not provided in the MES database. There is also a potential for recall bias as women’s responses regarding social support may have been influenced by their birth experiences or current circumstances, including their own health or their infant’s health. Furthermore, social support was not defined using a validated standardized tool. Despite the limitations, to our knowledge, no studies have examined the association of PPD and social support around pregnancy after adjusting for confounding variables and examined whether this relationship was similar for teen mothers and adult mothers in Canada.

## Conclusion

The present study amplified the need to identify mothers with low levels of support because they are at increased risk for depressive symptoms post partum. To develop effective interventions for mothers who are at more risk to PPD, it is essential to understand the relationship and role of social support during pregnancy and after birth. It is observed that social support after childbirth is important for both teen mothers and adult mothers to have, as it has been found to reduce the risk of experiencing PPD. Educating mothers on PPD soon after birth should be taken into account, as it acts as a protective factor.

## Abbreviations

CI: Confidence interval; EPDS: Edinburgh postnatal depression scale; MES: Maternity experiences survey; OR: Odds ratio; PPD: Postpartum depression; SPSS: Statistical package for social sciences.

## Competing interests

The authors declare that they have no competing interests.

## Authors’ contributions

THMK: Statistical analysis, literature review, and write-up of manuscript and critical revision for important intellectual content. JC: Contributed to the editing of the manuscript, and critical revision for important intellectual content. HT: Supervised analysis and critical revision for important intellectual content. All authors read and approved the final manuscript.

## Pre-publication history

The pre-publication history for this paper can be accessed here:

http://www.biomedcentral.com/1471-2393/14/162/prepub
